# Effect of blood ionised calcium concentration at calving on fertility outcomes in dairy cattle

**DOI:** 10.1136/vr.104932

**Published:** 2018-07-11

**Authors:** Philippa Jane Mahen, Helen J Williams, Robert Frank Smith, David Grove-White

**Affiliations:** Department of Livestock Health and Welfare, Institute of Veterinary Science, University of Liverpool, Liverpool, UK

**Keywords:** dairy cattle, fertility, herd health, hypocalcaemia

## Abstract

Clinical and subclinical hypocalcaemia are common in dairy cows, yet evidence in the literature assessing their impact on fertility is inconsistent. The aim of this prospective cohort study was to examine associations between blood ionised calcium concentration at calving and fertility outcomes in dairy cattle. Blood samples were taken from 137 Holstein cows from four commercial dairy herds within 24 hours of calving and analysed for blood ionised calcium using an Epocal Point of Care Analyser (Epocal, Ottawa, Canada). Data collected from routine veterinary fertility examinations and herd records were used to investigate the association of ionised calcium with the outcomes: time to first service, time to conception and endometritis. There were significant negative associations between blood ionised calcium concentration and time to first service (HR 1.33, P=0.001) and blood ionised calcium concentration and time to conception (HR 1.16, P=0.04). There was no significant association between blood ionised calcium concentration and endometritis. The results of this study imply that management policies that minimise the reduction in blood ionised calcium concentration in the periparturient dairy cow are likely to improve reproductive outcomes and should be considered as part of the multifactorial approach to optimising dairy cow fertility.

## Introduction

Periparturient dairy cows have a sudden increase in demand for calcium. The requirement for fetal growth in late gestation is only 10 g of calcium per day, but at calving this increases to 30–50 g/day for colostrum production.^1^ The homeostatic mechanisms controlling calcium concentration in the blood are often unable to respond quickly enough to meet this requirement. Consequently, clinical and subclinical hypocalcaemia are common in dairy cows.^2 3^ Clinical hypocalcaemia, or ‘milk fever’, shows a set of progressive signs; from initial lethargy and inappetence via hyperaesthesia and ataxia to recumbency, postural bloat and death.^4^ Cows with subclinical hypocalcaemia have none of the external signs of clinical hypocalcaemia but have low blood calcium concentration and may have disturbance of physiological function.^2^ Reported incidences of subclinical hypocalcaemia range from 15 to 50 per cent of periparturient cows,^5–11^ with higher incidence seen in multiparous cows.^7 9–11^ The majority of hypocalcaemic episodes in dairy cattle occur in the first 24 hours after calving.^6 12 13^

Within plasma, calcium exists in three forms: ionised, complexed and protein bound. Of these, ionised calcium (Ca^2+^) is the physiologically active form,^14^ therefore measurement of plasma Ca^2+^ concentration is a direct measure of physiologically available calcium, unlike total plasma concentration.

There are a number of putative mechanisms by which hypocalcaemia may affect bovine fertility including reduction in uterine contractility,^15–18^ increased risk of negative energy balance,^7 12 19^ suppressed immune function^19–21^ and decreased ovarian blood flow.^22^

Evidence in the literature as to whether hypocalcaemia has a clinically relevant detrimental effect on bovine fertility is limited and inconsistent. There are few studies on the impact of subclinical hypocalcaemia on fertility; more often fertility has been assessed in relation to clinical hypocalcaemia.^2 23^ Of these, some studies have found no association between clinical hypocalcaemia and reproductive outcomes.^24 25^ Other work has shown poorer reproductive outcomes in cows with clinical hypocalcaemia, including increased interval to first ovulation, longer luteal phase after the first ovulation,^16^ impaired ovarian cyclicity^17^ and increased times to both first service^26^ and conception.^26 27^ The smaller number of studies investigating the effects of subclinical hypocalcaemia also have varied outcomes. Chamberlin *et al*^12^ demonstrated no difference between hypocalcaemic and normocalcaemic groups in the percentage of cows cycling at 50–60 days post partum, services per conception or time to conception. Wilhelm *et al*^11^ found no difference in time to first service or pregnancies per artificial insemination between hypocalcaemic and normocalcaemic cows. In contrast, other studies have shown hypocalcaemic cows to have reduced ovarian cyclicity at 49 days^8^ and be less likely to become pregnant to the first service.^28^ Caixeta *et al*^29^ found that cows that exhibited hypocalcaemia over three consecutive days post partum (‘chronic subclinical hypocalcaemia’) were less likely to return to cyclicity and to become pregnant to the first service.

The aim of the present study was to examine the effect of blood Ca^2+^ concentration in the immediate postpartum period on subsequent fertility in dairy cattle.

## Materials and methods

The study design was reviewed and approved by the University of Liverpool Veterinary Research Ethics Committee prior to commencement of research (reference VREC318). Research was carried out under Project Licence 40/3478 issued by the UK Home Office under the Animals (Scientific Procedures) Act 1986.

Cows were recruited from four commercial year-round calving Holstein dairy herds in north-west England. Herds had a routine veterinary fertility visit at least once per fortnight which included, as a minimum, examination of all cows at 21–35 days after calving, pregnancy diagnosis at 28–49 days after service and examination of cows presented by the farmer as not observed in oestrus. All cows were served using artificial insemination. For each herd, fertility data were recorded on a database (*Interherd*, PAN Livestock Services, Reading). This included parity, calving date and whether assistance was given, service dates, pregnancy diagnosis results and results of fertility examinations, including presence of endometritis.^30^ The milk yield of each cow in the herd was downloaded from the parlour software monthly by a technician (National Milk Records, Chippenham or Cattle Information Service, Rickmansworth). This information was imported into the herd database (*Interherd*, PAN Livestock Services) which provided an estimate of 305-day milk yield for each cow.

All calving cows and heifers were eligible to be recruited onto the study unless the calving was deemed an abortion (fetus expelled prior to 271 days’ gestation^31^) or the animal had been treated with a calcium containing product prior to blood sampling, either since calving or in the 24 hours prior to calving.

Farmers were asked to notify researchers, by telephone or text message, as soon as was practicable after an eligible cow had calved so that a visit could be made within 24 hours of calving. At the visit, a blood sample was taken from the coccygeal vein into a 6 ml lithium heparin-coated *Vacutainer* (BD, Franklin Lakes, USA). As soon as possible after sample collection an aliquot of blood was removed from the *Vacutainer* using a syringe and entered into an Epocal Point of Care Analyser (Epocal, Ottawa, Canada) for measurement of Ca^2+^ by selective electrode potentiometry.

The following information was recorded at the time of blood sampling: date and time of blood sampling, date and time of calving and body condition score (BCS).^32^ Farmers were free to administer calcium-containing products after blood sampling, according to clinical need or on-farm protocols; these administrations were recorded by the farmer. Blood sampling took place between June 2015 and June 2016. Cows remained in the study until either they successfully conceived or they exited through death, sale from the herd, being designated a non-breeding animal by the farmer or reaching the end of the study period (March 31, 2017, ie, a minimum of nine months after blood sampling).

Data were collated in a spreadsheet (*Excel 2013*, Microsoft) and imported into Stata V.11 (StataCorp, College Station, USA) for statistical analysis. For ease of interpretation Ca^2+^ values were multiplied by 10 before analyses were performed. Parity was categorised as one, two, three and ‘four or higher’. BCS was categorised as less than 2.75, 2.75–3.25 and more than 3.25, and 305-day milk yield was divided into terciles. The variable ‘Season of calving’ was created (where March to May=spring, June to August=summer, September to November=autumn and December to February=winter).

A multivariable linear regression model was fitted with blood Ca^2+^ concentration as the dependent variable. The variable ‘farm’ was forced into the model. Other initial covariates offered to the model were: time from calving to sampling, parity, BCS category, 305-day yield tercile and season. A backward stepwise model-building strategy using the likelihood ratio test was employed, taking P<0.2 for retention of a variable in the final model.

Reproductive outcomes assessed were time to first service, time to conception and occurrence of endometritis.

The association between time to first service or time to conception and blood Ca^2+^ concentration was investigated using a Cox proportional hazards model. The data were right censored; cows that exited the study before undergoing the event in question (first service or conception) were deemed censored and the days between calving and censoring recorded. Blood Ca^2+^ concentration and farm were both forced into the model. Other covariates included in the initial model were: parity, BCS category, 305-day yield tercile, season, assistance at calving, whether calcium was administered after sampling and endometritis. A backward stepwise model-building strategy using the likelihood ratio test was employed, taking P<0.2 for retention of a variable in the final model.

The association between blood Ca^2+^ concentration and endometritis was assessed using multivariable logistic regression. A backward stepwise approach to selection of explanatory variables was employed, as outlined previously. Blood Ca^2+^ concentration and farm were forced into the model. Other covariates included in the initial model were: parity, BCS category, 305-day yield tercile, season, assistance at calving and whether calcium was administered after sampling.

In all analyses, results were considered significant at P<0.05.

## Results

One hundred and thirty-seven cows were enrolled in the study, the characteristics of the study population are shown by farm in Table 1. The median parity of study cows was 2 (IQR 1–3) and the mean 305-day milk yield of the previous lactation (for cow parity 2 or above) was 9286 litres (sd 2317 litres). Exogenous calcium was administered to 36/137 (26 per cent) cows subsequent to the blood sample being obtained; the majority of these cows were on farms 1 and 3 as they had policies of administering oral calcium boluses after calving to all cows of parity 3 or above. Of the 137 animals included in the study, 125 (91 per cent) received a first service. Twenty-two cows exited the breeding herd before conceiving, one cow reached the end of the study period without having conceived, the remaining 114 (83 per cent) cows conceived.

**TABLE 1:** Summary statistics of study population

Farm	1	2	3	4	Total
**Number of cows enrolled in study**	58	45	12	22	137
**Parity** Median (range)	2 (1–8)	2 (1–6)	2 (1–2)	3 (1–6)	2 (1–8)
**305-day yield last lactation if applicable (litres)** Mean (sd)	8999 (1831)	8757 (1908)	6806 (1378)	12,142 (1627)	9286 (2317)
**Body condition score** Median (range)	2.75 (2.25–3.5)	3 (2.25–3.5)	3.125 (3–3.75)	3.25 (2.5–3.5)	3 (2.25–3.75)
**Cows assisted at calving** Number of cows (%)	16 (28)	16 (36)	4 (33)	4 (18)	40 (29)
**Cows that received calcium after sampling** Number of cows (%)	1 (2)	23 (51)	0 (0)	12 (55)	36 (26)
**Voluntary waiting period (days)**	Parity 1 animals 70, others 50	60	42	50	NA
**Endometritis** Number of cows (%)	11 (19)	13 (29)	6 (50)	4 (18)	34 (25)
**Cows exiting before conception** Number of cows (%)	8 (14)	10 (22)	2 (17)	2 (9)	22 (16)
**Blood ionised calcium concentration (mmol/l)** Mean (range)	1.04 (0.53–1.32)	0.98 (0.55–1.22)	1.12 (1.00–1.23)	1.02 (0.69–1.23)	1.02 (0.53–1.32)
**Time between calving and blood sampling (minutes)** Mean (range)	640 (20–1433)	624 (2–1380)	694 (3–1440)	416 (20-825)	603 (2–1440)

NA, not applicable.

**Figure 1:**
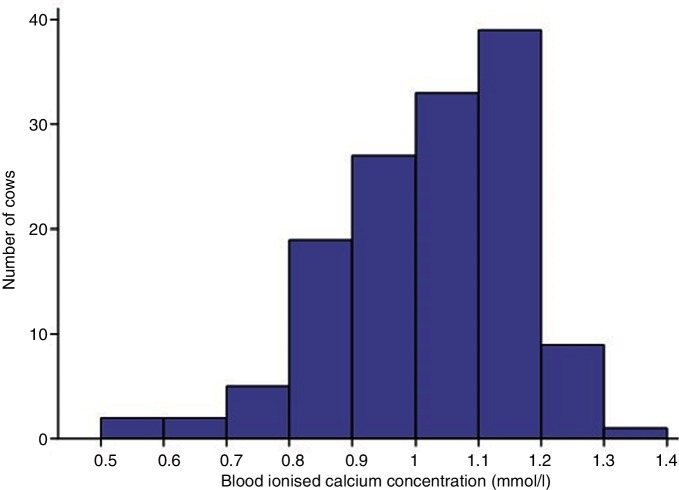
Histogram of blood ionised calcium concentrations obtained in cows in the first 24 hours after calving (n=137).

Blood Ca^2+^ concentrations obtained in the first 24 hours after calving ranged from 0.53 to 1.32 mmol/l with a median of 1.06 mmol/l (figure 1). Following backward stepwise elimination, the final linear regression model (Table 2) suggested that Ca^2+^ concentration was associated (P<0.05) with season of calving, BCS and parity.

**TABLE 2:** Multivariable linear regression model for blood ionised calcium concentration (mmol/lx10) in the first 24 hours after calving

Variable	Coefficient	95% CI	P values
**Farm** (Baseline: Farm 1)			
Farm 2	−1.00	−1.50 to −0.50	<0.001
Farm 3	0.39	−0.49 to 1.26	0.38
Farm 4	−0.27	−0.91 to 0.38	0.41
**Season** (Baseline: Spring)			
Summer	0.15	−0.44 to 0.74	0.62
Autumn	0.07	−0.55 to 0.68	0.83
Winter	−0.80	−1.38 to −0.21	0.01
**Body condition score category** (Baseline: <2.75)			
2.75–3.25	0.39	−0.14 to 0.92	0.14
>3.25	−0.28	−1.16 to 0.59	0.53
**Parity** (Baseline: 1)			
2	−0.96	−1.51 to −0.41	0.001
3	−1.14	−1.72 to −0.56	<0.001
4 or higher	−2.35	−2.88 to −1.83	<0.001
Constant	11.49	10.87 to 12.12	

An increase in blood Ca^2+^ concentration was associated with a reduction in the time to first service (HR 1.33 per 0.1 mmol/l change in Ca^2+^, P=0.001). Increased parity was also associated with a reduced time to first service, while presence of endometritis was associated with an increased time to first service (Table 3).

**TABLE 3:** Cox proportional hazards models for time to first service and time to conception

	HR	95% CI	P values
**Time to first service**			
**Ca^2+^ (per 0.1 mmol/l change)**	1.33	1.12 to 1.57	0.001
**Farm** (Baseline: Farm 1)			
Farm 2	1.06	0.69 to 1.63	0.79
Farm 3	2.47	1.15 to 5.33	0.02
Farm 4	1.37	0.80 to 2.36	0.25
**Parity** (Baseline: 1)			
2	1.45	0.83 to 2.55	0.19
3	1.46	0.81 to 2.65	0.21
4 or higher	3.23	1.60 to 6.52	0.001
**Endometritis**	0.65	0.42 to 0.98	0.04
**Time to conception**			
**Ca^2+^ (per 0.1 mmol/l change)**	1.16	1.01 to 1.33	0.04
**Farm** (Baseline: Farm 1)			
Farm 2	0.88	0.56 to 1.38	0.58
Farm 3	1.42	0.69 to 2.92	0.34
Farm 4	0.84	0.50 to 1.42	0.52
**Assistance at calving**	0.70	0.46 to 1.05	0.09
**Endometritis**	0.67	0.41 to 1.07	0.09

An increase in blood Ca^2+^ concentration was associated with a reduction in the time to conception (HR 1.16 per 0.1 mmol/l change in Ca^2+^, P=0.04). Assistance at calving and presence of endometritis were associated with an increase in time to conception (Table 3).

There was no association between blood Ca^2+^ concentration at calving and the likelihood of a cow being diagnosed with endometritis (OR 1.22, P=0.29) (Table 4). However, administration of calcium after calving was associated with a decreased likelihood of endometritis being diagnosed. Calving during the summer months was associated with a greater likelihood of endometritis.

**TABLE 4:** Multivariable logistic regression model for endometritis

	OR	95% CI	P values
**Ca^2+^ (per 0.1 mmol/l change)**	1.22	0.84 to 1.77	0.29
**Farm** (Baseline: Farm 1)			
Farm 2	1.85	0.57 to 6.02	0.31
Farm 3	1.60	0.37 to 6.97	0.54
Farm 4	0.79	0.17 to 3.58	0.76
**Calcium administration after calving**	0.38	0.10 to 1.44	0.16
**Season** (Baseline: Spring)			
Summer	5.51	1.24 to 24.37	0.03
Autumn	1.87	0.35 to 10.11	0.47
Winter	2.93	0.63 to 13.56	0.17

## Discussion

This prospective cohort study investigated associations between blood Ca^2+^ concentration after calving and reproductive outcomes in dairy cattle. Blood samples were taken within the first 24 hours post partum, since previous studies demonstrated that the majority of hypocalcaemic episodes in dairy cattle occur in this period.^6 12 13^ The point in the 24-hour period at which cows were sampled had no significant association with blood Ca^2+^ concentration. Being of higher parity was a significant risk factor for lower blood Ca^2+^ concentration, in agreement with previous studies.^7 9–11^

There was a significant association between blood Ca^2+^ concentration in the first 24 hours after calving and time to first service. For every 0.1 mmol/l increase in blood Ca^2+^ there was a 33 per cent increase in the chance of a cow being served at any given point in time (HR 1.33, P=0.001). There was also a significant association between blood Ca^2+^ concentration and time to conception. For every 0.1 mmol/l increase in Ca^2+^ there was a 16 per cent increase in the chance of a cow conceiving at any given point in time (HR 1.16, P=0.04). Interestingly, these associations remained true for increases in blood Ca^2+^ concentrations at the higher end of the range, indicating that there may be scope to improve fertility in cows previously considered to be normocalcaemic as well as increasing fertility by preventing subclinical hypocalcaemia. Therefore, management interventions in the dry period aimed at increasing blood calcium concentrations across the range, rather than aimed solely at hypocalcaemic cows, will likely offer most fertility benefits.

In contrast to the present study, Wilhelm *et al*^11^ and Chamberlin *et al*^12^ found no association between blood calcium concentration and time to first service or time to conception, respectively. However, in both cases calcium concentration was considered as a binary variable, with cows classified as hypocalcaemic or normocalcaemic, rather than a continuous variable as in the present study. Additionally, Wilhelm *et al*^11^ considered total serum calcium concentration as opposed to Ca^2+^ concentration which can be affected by blood pH and protein concentrations.

No significant association was found between Ca^2+^ and endometritis. This is in agreement with Chapinal *et al*^33^ and Chamberlin *et al*,^12^ but in contrast to Martinez *et al*^19^ who found increased incidence of metritis in cows with subclinical hypocalcaemia. This may represent a genuine lack of association or it may be that the incidence of endometritis in the study population (25 per cent) meant that statistical power was insufficient to detect an association. Martinez *et al*^19^ was notable for a higher incidence of metritis within the study population (47.3 per cent) compared with the studies where no significant association was found (Chapinal *et al*,^33^ 16.7 per cent; Chamberlin *et al*,^12^ 8.0 per cent).

The poorer fertility parameters seen in cows with lower blood concentrations of calcium could be due to impairment of physiological processes involved in return to ovarian cyclicity, expression of oestrus and ability to conceive and maintain a pregnancy. Putative mechanisms by which this could occur include increased negative energy balance in cows with lower calcium,^7 12 19^ reduced ovarian blood flow,^22^ reduction in immune function^19 20^ and reduction in uterine contractility, and hence involution in cows with lower calcium.^15–18^ Evaluation of any association between Ca^2+^ concentration and the physiological processes occurring between calving and conception would be a useful direction for future research.

A proportion of cows in the study population received calcium boluses (after blood sampling) due to on-farm protocols and therefore administration of calcium was offered as a variable to the statistical models. It did not remain in the models for time to first service and time to conception. It remained in the model for endometritis but at low significance (Table 4). It is not possible to conclude from this study whether or not administration of calcium boluses has any impact on reproductive outcomes, therefore bolus administration after calving may not be a substitute for prevention. A previous randomised controlled trial by Oetzel and Miller^34^ found no difference in time to conception between bolus-treated and untreated groups.

In conclusion, there is a negative association between blood Ca^2+^ concentration in the first 24 hours after calving and both time to first service and time to conception. This implies that management policies that minimise the reduction in blood Ca^2+^ concentration in the periparturient dairy cow are likely to improve reproductive outcomes. Farmers and veterinary surgeons should consider the impact of periparturient blood calcium concentration as part of their multifactorial approach to optimising dairy herd fertility.
